# HOW ARE SOCIAL ISOLATION AND LONELINESS RELATED TO COGNITIVE FUNCTIONING AMONG CHINESE OLDER ADULTS?

**DOI:** 10.1093/geroni/igz038.3129

**Published:** 2019-11-08

**Authors:** Rumei Yang, Haocen Wang, Eunjin L Tracy, Linda S Edelman, Katherine Sward, George Demiris, Gary Donaldson

**Affiliations:** 1 School of Nursing Nanjing Medical University, Nanjing, Jiangsu, China; 2 University of Wisconsin-Madison, Madison, Wisconsin, United States; 3 University of Utah, SLC, Utah, United States; 4 University of Utah, Salt Lake City, Utah, United States; 5 University of Utah College of Nursing, SLC, Utah, United States; 6 University of Pennsylvania School of Nursing, Philadelphia, Pennsylvania, United States; 7 University of Utah School of Medicine, SLC, Utah, United States

## Abstract

Older adults have increased risk of social isolation, loneliness, and cognitive functioning decline, but the relationships among these factors are not conclusive. We used the 2011 and 2012 waves of the harmonized China Health and Retirement Longitudinal Study to: 1) measure the association between social isolation and cognitive functioning among Chinese older adults within their cultural context, and 2) investigate the potential mediation mechanism of loneliness on this association. Specifically, we applied a multiple indicator multiple cause approach to determine whether the construct of social isolation is well defined by four indicators (social activity engagement, weekly adult children contact, caregiving for grandchildren, and living alone). We used structural equation modeling to examine the direct and indirect effects among variables of interest. The results demonstrated that three indicators of social isolation were significantly associated with cognitive functioning (β =-0.26 to -0.28, all ps<0.05). The indirect effect of social isolation on cognitive functioning through loneliness was significant (β = -0.15, p<0.05), indicating loneliness was an important mediator. After controlling for the indirect effect of loneliness, the direct effect of social isolation on cognitive functioning remained significant (β =-0.83, p<0.05), suggesting a partial mediation effect. Our study confirms that social isolation contributes to cognitive functioning decline among Chinese older adults and that loneliness plays a mediating role. The findings suggest maintaining social relations and coping with feelings of loneliness are beneficial to older adults’ cognitive functioning.

